# Complications of Bacterial Keratitis Impacted by Social Determinants of Health: A Case Report

**DOI:** 10.5811/cpcem.1594

**Published:** 2024-01-25

**Authors:** Taylor Diederich, Mary Velagapudi, Hope Ring

**Affiliations:** Department of Emergency Medicine, University of Kansas Medical Center, University of Kansas Health System, Kansas City, Kansas

**Keywords:** *keratitis*, *vision loss*, *hypopyon*, *corneal ulcer*, *pain*

## Abstract

**Introduction:**

The emergency department commonly evaluates eye pain and vision loss. Typically, these conditions can be managed outpatient; however, delays can lead to advanced pathology.

**Case Report:**

A 48-year-old homeless male presented with left-eye vision loss and pain. His exam revealed monocular decreased visual acuity, corneal ulcer, and hypopyon. The patient was diagnosed with bacterial keratitis and admitted for treatment but left against medical advice. He returned and was admitted for further treatment but was lost to follow-up thereafter.

**Conclusion:**

Our case features complicated bacterial keratitis with several treatment interruptions, demonstrating how healthcare disparities contribute to potentially preventable advanced pathology.

Population Health Research Capsule What do we already know about this clinical entity?

*Bacterial keratitis is an ophthalmologic infection; if untreated it can progress to complications, some of which cause permanent vision loss.*

What makes this presentation of disease reportable?

*Emergency Department eye complaints are common. Understanding of complications and the ways that health disparities affect outcomes is important for physicians.*

What is the major learning point?

*Treatment of bacterial keratitis is paramount to prevention of life-altering complications. Social factors can strongly influence disease course and management.*

How might this improve emergency medicine practice?

*Emergency physicians must understand serious implications of bacterial keratitis and must consider social barriers to care.*

## INTRODUCTION

Ophthalmologic complaints are common in the emergency department (ED).^1^ Pain and vision loss are among the most common chief complaints. Although eye infections are commonplace, advanced bacterial disease is uncommon.^2^ In this report, we discuss the case of a man who suffered infectious bacterial keratitis but delayed presentation to care until the presence of a severe corneal ulcer with hypopyon threatened his vision. We speculate that his delay in presentation was complicated by use of contact lenses, medication theft, and barriers imposed by healthcare disparities.

## CASE REPORT

**Image 1.**
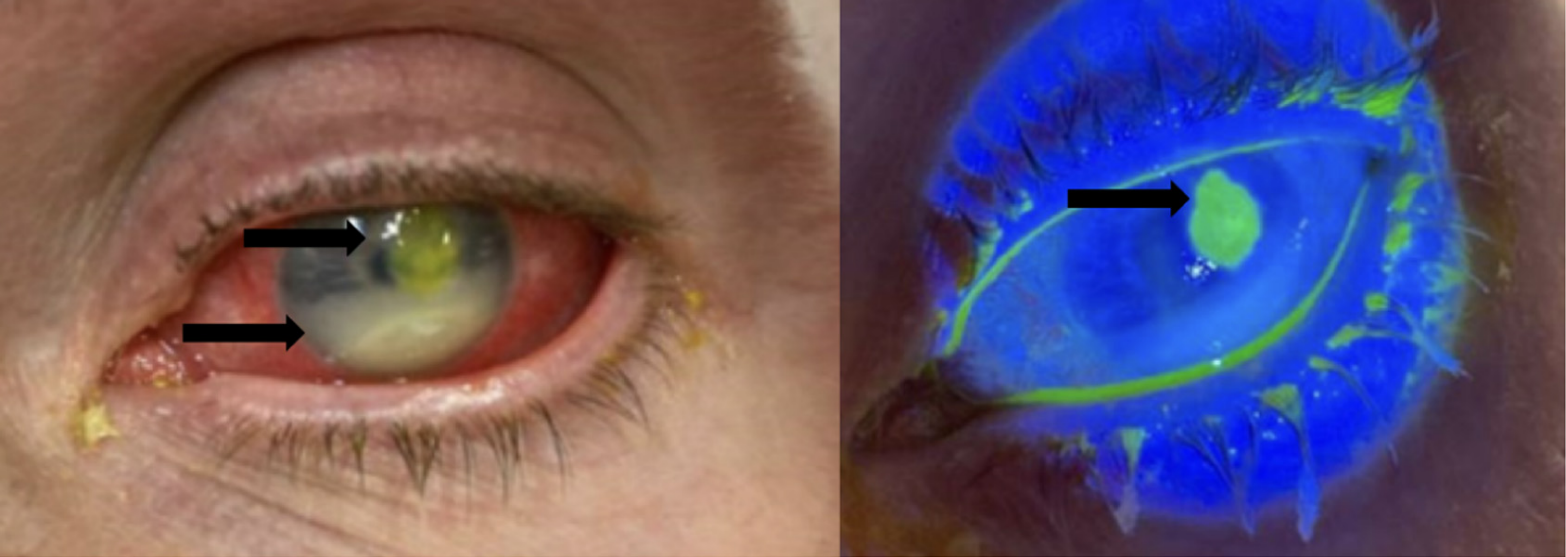
Left eye on initial presentation under normal lighting (left) and Woods lamp (right), demonstrating conjunctival injection, hypopyon, and fluorescein-stained paracentral corneal ulcer. Top arrow in the left image points to fluorescein-stained corneal ulcer, while the bottom arrow in the left image points to hypopyon. The arrow in the right image points to fluorescein-stained corneal ulcer.

A 48-year-old homeless male presented to the ED with left-eye vision loss and pain. The patient described the gradual onset of these symptoms over three weeks, causing him to stop wearing his contact lenses two weeks prior to presentation. The patient endorsed a similar problem in the past that resolved with the use of eye drops. He also noted that the center of his eye had begun to appear white. The patient denied fever, chills, nausea, vomiting, and headache. Upon initial evaluation, the patient demonstrated 20/25 vision bilaterally, 20/30 vision in his right eye, and only finger counting at approximately two feet in his left eye. Extraocular movements were intact; intraocular pressures were 13 millimeters of mercury (mm Hg) in the right and 24 mm Hg in the left (reference range 10–21 mm Hg).^3^ Bilateral pupils were reactive to light without afferent pupillary defect. By visual examination, the patient demonstrated prominent conjunctival injection, a 4 mm × 4 mm round corneal ulcer in the three o’clock position of the cornea, and a hypopyon covering one third of the anterior chamber with a line of sediment covering one half the anterior chamber (reaching a height of five mm). On fluorescein staining, the corneal ulcer appeared yellow without any other dye uptake or Seidel sign (Image 1).

Computed tomography (CT) of the orbits demonstrated asymmetric decrease in volume and enhancement along the left anterior chamber, without evidence of posterior inflammation or retrobulbar abscess. Ophthalmology was consulted but could not attain a dilated retinal exam on the left due to overlying anterior chamber opacity. B-scan ultrasound was performed without evidence of endophthalmitis. Ophthalmology collected corneal cultures, and the patient was admitted for monitoring and administering topical vancomycin, tobramycin, and atropine drops. Unfortunately, the patient left against medical advice (AMA) two days later; he stated that this choice was due to difficulty with his insurance. The corneal culture collected on admission ultimately grew *Staphylococcus epidermidis* with resistance to clindamycin and erythromycin.

**Image 2.**
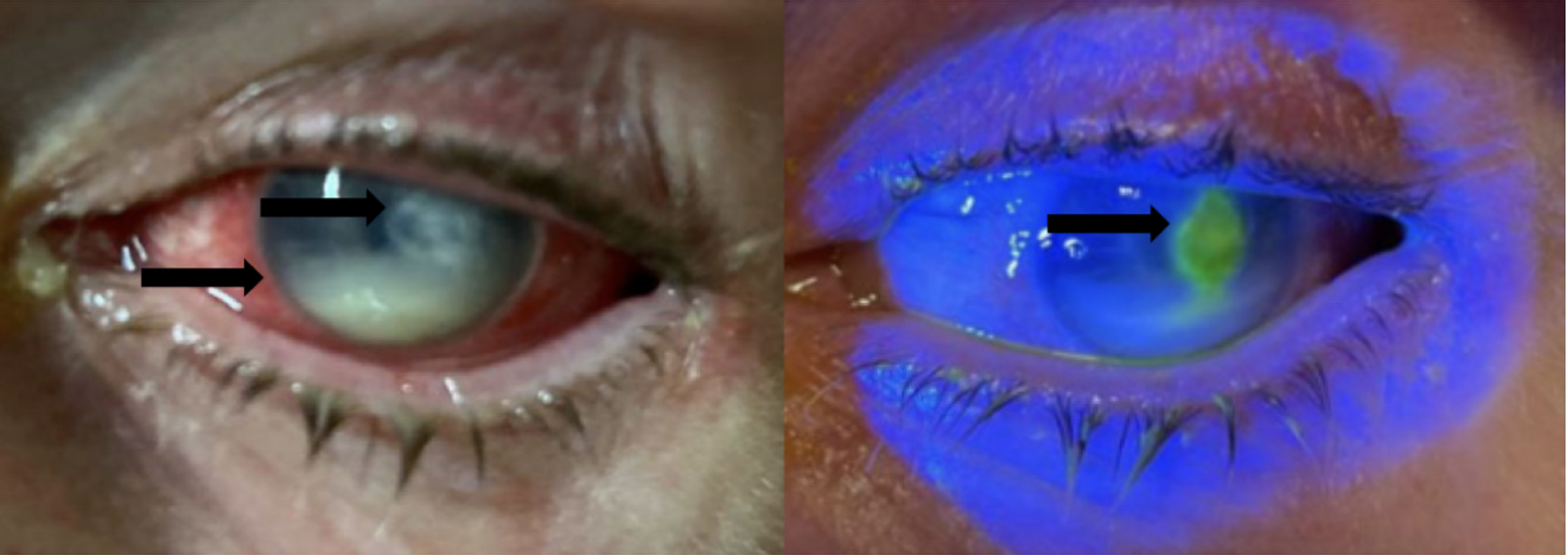
Left eye on second emergency department presentation under normal lighting prior to fluorescein staining (left) and under Woods lamp after fluorescein administration (right), demonstrating conjunctival injection, hypopyon, and paracentral corneal ulcer with fluorescein uptake. Top arrow in the left image points to unstained corneal ulcer, while the bottom arrow in the left image points to hypopyon. The arrow in the right image points to fluorescein-stained corneal ulcer.

The patient returned five days later due to concern for worsening symptoms. He reported good compliance to his vancomycin drops six times per day and tobramycin drops twice daily. He demonstrated 20/20 vision in his right eye but only hand motions with light perception in his left eye, which was worse than his first exam. Extraocular movements remained intact. Pupils were reactive to light bilaterally without afferent pupillary defect. Visual examination yielded continued conjunctival injection, enlarged 6 mm x 4 mm round corneal ulcer at three o’clock, and similar hypopyon covering one third to one half of the anterior chamber (Image 2).

**Image 3.**
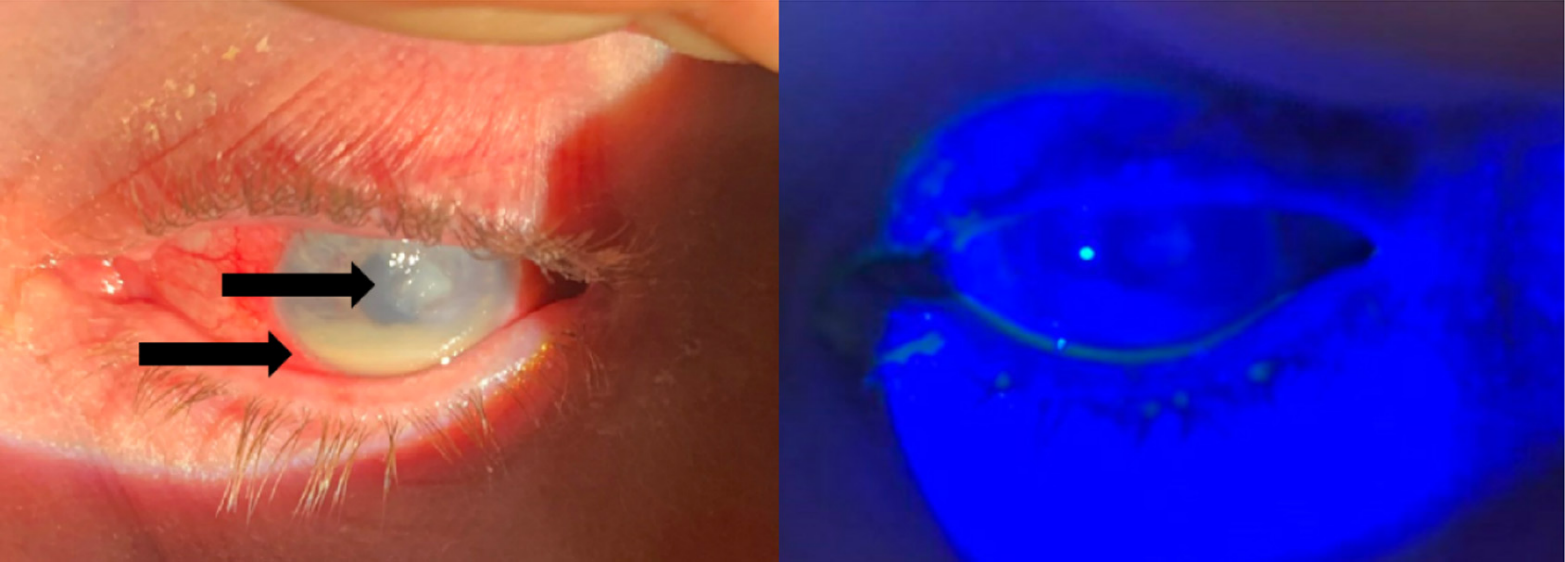
Left eye on the day of discharge under normal lighting prior to fluorescein staining (left) and under Woods lamp after fluorescein administration (right), demonstrating conjunctival injection, reduction in the size of hypopyon, and paracentral corneal ulcer without fluorescein uptake under Woods lamp. The top arrow in the left image points to an unstained corneal ulcer, while the bottom arrow in the left image points to hypopyon.

Ophthalmology was consulted, and corneal cultures were re-collected. He was admitted for continued vancomycin, tobramycin, and atropine drops. Repeat corneal cultures grew *Cutibacterium acnes* and *Psychrobacter faecalis/pulmonis.* The patient remained inpatient for six days with improved sight and pain control. At this point, he was noted to show a reduction in the size of the hypopyon and severity of the ulcer (Image 3).

He was then discharged to continue vancomycin and atropine drops for one week and follow up with ophthalmology. Unfortunately, the patient returned to the ED six days after discharge reporting that his medications had been stolen from him; he was provided with refills and discharged from the ED.

## DISCUSSION

Infectious keratitis is defined as an infection of the cornea, which is the anterior-most layer of the eye. These infections can be due to bacteria, viruses, fungi, and protozoa; they are commonly polymicrobial. Most causes of infectious keratitis are bacterial, *Pseudomonas* and *Staphylococci* species being most common.^4^ ^,^ ^5^ Of note, *Staphylococcus* and *Streptococcus* are considered normal flora of the eye, but are also possible culprits.^6^ Patient typically present within 24 hours of infectious keratitis development, complaining of eye pain, photophobia, and vision changes. Although diagnosis of the culpable organism can be made by corneal culture, this is not typically considered necessary.

Although readily treatable, infectious keratitis can progress to more serious complications. These complications include corneal scarring, corneal anesthesia, perforation, secondary glaucoma, hyphema, hemorrhage, lens subluxation, anterior subcapsular cataract, corneal fistula, scleritis, retinal detachment, choroidal detachment, endophthalmitis, panophthalmitis, keratectasia, atrophic bulbi, autoevisceration, and retrobulbar abscess.^4^ ^–^ ^8^ Corneal ulcer—thinning and compromised integrity of the cornea—can occur with severe infectious keratitis. Clinically, one may distinguish ulceration from keratitis by fluorescein uptake. When severe, it may lead to corneal perforation.^8^

Hypopyon, on the other hand, may occur with or without accompanying ulceration in the setting of infectious keratitis and involves layering of inflammatory cell milieu in the anterior chamber.^8^ This appears clinically as an opaque, white layer within the bottom of the anterior chamber. This is typically visualized easily on physical exam, with better definition via slit lamp.

Another rare albeit serious complication of infectious keratitis is endophthalmitis—infection of the anterior and posterior chambers without spreading exteriorly (ie, no scleral involvement). This typically occurs in advanced keratitis leading to corneal perforation, and it may progress rapidly.^5^ ^,^ ^7^ ^–^ ^9^ Panophthalmitis is an extension of infection to sclera and exterior eye structures, such as the eyelid. This can be an especially severe complication since the appearance mimics cellulitis; thus, underlying infection is more difficult to detect. Exam findings can include chemosis, proptosis, and eyelid edema. These are probably the most serious potential complications in that they may lead to permanent vision loss without rapid treatment.^9^ In 2022, Zeng et al demonstrated a progression rate of 9.46% from bacterial keratitis with hypopyon to endophthalmitis; in this study, a hypopyon of greater than 3 mm was found to demonstrate a 4.12 odds ratio of eventual endophthalmitis.^10^

Even when treated appropriately and complications do not occur, the scarring associated with central or paracentral infection can cause substantial loss of vision.^5^ Workup of suspected infectious keratitis and its associated complications initially involves slit lamp exam, measurement of intraocular pressure (IOP), and fluorescein staining.^5^ These measures should be sufficient to diagnose and initiate appropriate treatment for infectious keratitis, corneal ulceration, corneal perforation, and hypopyon. If concern exists for corneal ulcer, perforation, or posterior spread of infection, ophthalmology should be consulted. When endophthalmitis, panophthalmitis, or retrobulbar abscess are suspected, imaging with ultrasound and/or CT of the orbits is indicated. Clinical factors that may raise suspicion for these entities include sepsis, preceding intraocular surgery, and penetrating trauma; unfortunately, the symptoms and presenting signs overlap significantly with the above alternative diagnoses; therefore, one must keep a high index of suspicion.^5^ ^,^ ^9^ Regarding retrobulbar abscess, proptosis and/or increased IOP should raise a red flag to prompt further imaging.

Contact lens use is a major cause of bacterial keratitis.^2^ ^,^ ^4^ ^–^ ^6^ ^,^ ^8^ ^,^ ^11^ Various factors contribute, including prolonged lens use, inadequate cleaning, using tap water as a cleaning agent, contaminated solution, and contact lens trauma.^5^ Our patient’s history of substance use and homelessness may have negatively impacted his hygiene.

Initial antibiotic therapy, as in our patient, includes a dual-agent topical regimen (vancomycin and tobramycin). Other options include monotherapy with a fourth-generation fluoroquinolone.^4^ ^–^ ^6^ Cycloplegics are also indicated for pain control as they prevent ciliary muscle constriction.^5^ ^,^ ^6^ ^,^ ^8^ Hourly administration of eye drops is necessary for the first 24–48 hours.^6^ ^,^ ^8^ Daily eye examinations are indicated for a minimum of three days, and treatment typically continues for at least two weeks.^6^ Since there were concerns about our patient’s ability to follow up on an outpatient basis, he was admitted for six days; however, most individuals may seek outpatient care for the entirety of their course.

The prognosis of bacterial keratitis is governed by a multitude of factors. Ulcer confinement superficial to the middle third of the stroma, which is the corneal layer between the outer and inner epithelia, portends a favorable prognosis. Deeper ulcers and those involving visual axis, stromal melt, and corneal thinning tend to fare worse. Treatment and follow-up access and adherence also play into overall prognosis.^8^

In 2021, Ting et al found poor visual outcomes with delayed healing correlated to the following risk factors at presentation: age >50 years; infiltrate size greater than 3 mm; and reduced visual acuity.^11^ Our patient demonstrated two of these risk factors, in addition to the unstudied but likely substantial risk factors of homelessness, inability to pursue outpatient follow-up, and inability to comply with outpatient medical therapy. Notable complications revealed in this retrospective review included glaucoma, recurrent infection, loss of vision, corneal perforation, and enucleation; 16.3% of patients in this population were found to require surgical intervention.^11^

In this case, social factors adversely affected this patient’s course. The patient presented at an advanced stage of bacterial keratitis, with complications compromising his vision. Homelessness and poor access to healthcare likely weighed heavily into the decisions made by these physicians. For example, physicians favored admission, whereas outpatient management might have otherwise been pursued. In cases such as these, emergency clinicians must also ask themselves how they can address the driving forces. As one example, might this patient have benefited from housing resources, given the theft of his medications on the street? We must remember it is within our scope to connect patients to social resources and make moves within the system to make preventative care more accessible to underserved patients.

## CONCLUSION

Bacterial keratitis is a significant cause for vision loss. This presentation with corneal ulcer, hypopyon, and compromised visual acuity starkly demonstrates the adverse effects of healthcare disparities. Our patient presented to care with advanced pathology and went on to fail inpatient and outpatient therapy, putting him at risk for further life-altering complications. One can envision the interplay of improper contact lens care, homelessness, and poor healthcare access contributing to his complications. It reinforces the importance of the ED’s role in making this diagnosis and escalating care. We are also the primary point of contact for vulnerable individuals; thus, we must address healthcare disparities as they relate to the acute presentation and ongoing underlying threat to our patients’ health.
